# Clinical usefulness of microsatellite instability for the prediction of gastric adenoma or adenocarcinoma in patients with chronic gastritis

**DOI:** 10.1054/bjoc.1999.1154

**Published:** 2000-06

**Authors:** K Kashiwagi, M Watanabe, T Ezaki, T Kanai, H Ishii, M Mukai, T Hibi

**Affiliations:** Department of Internal Medicine, School of Medicine, Keio University; Keio Cancer Center, 35 Shinanomachi, Shinjuku-ku, Tokyo, 160-8582, Japan; Department of Pathology, School of Medicine, Keio University, Tokyo, Japan

**Keywords:** microsatellite instability, gastritis, gastric adenoma, gastric adenocarcinoma, *Helicobacter pylori*

## Abstract

To assess a role of microsatellite instability (MSI) in the development of gastric adenocarcinoma or adenoma from chronic gastritis, we analysed mutations of five microsatellite loci in gastritis, adenoma and adenocarcinoma retrospectively. Gastric mucosa was biopsied from the same area in each patient at different periods and examined for MSI. Only one of 55 patients with chronic gastritis revealed MSI-H phenotype and the other 54 patients showed microsatellite stable (MSS) phenotypes. In six of 17 patients with gastric adenoma or well-differentiated adenocarcinoma, MSI-positive phenotypes were demonstrated. Interestingly, all of six patients showing MSI, including three high-level MSI (MSI-H) cases and three low-level (MSH-L) cases, had already revealed MSI at the stage of chronic gastritis. In two of three MSI-H cases, the identical MSI patterns had been observed at the stage of gastritis 1.5–7 years before the final diagnosis of adenocarcinoma. The adjacent gastritis mucosa within 10 mm from the carcinoma demonstrated MSI as well. MSI was not found in any of 35 patients with *Helicobacter pylori* infection, but found in one of 30 patients without infection. Moreover, two of three cases of gastric adenoma or well-differentiated adenocarcinoma with MSI-H at the stage of chronic gastritis showed no evidence of *Helicobacter pylori* infection throughout the observation periods. These results indicate that MSI in biopsy specimens at the stage of chronic gastritis may predict the risk of the progression to adenoma and well-differentiated adenocarcinoma, and that *Helicobacter pylori* infection itself may not induce MSI directly in the gastric mucosa. © 2000 Cancer Research Campaign


*British Journal of Cancer* (2000) 82(**11**), 1814–1818
© 2000 Cancer Research Campaign
DOI: 10.1054/ bjoc.1999.1154, available online at http://www.idealibrary.com on IDEAL®

### Clinical usefulness of microsatellite instability for the prediction of gastric adenoma or adenocarcinoma in patients with chronic gastritis

**K Kashiwagi[1], M Watanabe[2], T Ezaki[1], T Kanai[1], H Ishii[1], M Mukai[3] and T Hibi[1,2]**

[1]Department of Internal Medicine, School of Medicine, Keio University; [2]Keio Cancer Center , 35 Shinanomachi, Shinjuku-ku, Tokyo 160-8582, Japan; [3]Department of Pathology, School of Medicine, Keio University, Tokyo, Japan

**Summary** To assess a role of microsatellite instability (MSI) in the development of gastric adenocarcinoma or adenoma from chronic gastritis, we analysed mutations of five microsatellite loci in gastritis, adenoma and adenocarcinoma retrospectively. Gastric mucosa was biopsied from the same area in each patient at different periods and examined for MSI. Only one of 55 patients with chronic gastritis revealed MSI-H phenotype and the other 54 patients showed microsatellite stable (MSS) phenotypes. In six of 17 patients with gastric adenoma or well-differentiated adenocarcinoma, MSI-positive phenotypes were demonstrated. Interestingly, all of six patients showing MSI, including three high-level MSI (MSI-H) cases and three low-level (MSH-L) cases, had already revealed MSI at the stage of chronic gastritis. In two of three MSI-H cases, the identical MSI patterns had been observed at the stage of gastritis 1.5–7 years before the final diagnosis of adenocarcinoma. The adjacent gastritis mucosa within 10 mm from the carcinoma demonstrated MSI as well. MSI was not found in any of 35 patients with *Helicobacter pylori* infection, but found in one of 30 patients without infection. Moreover, two of three cases of gastric adenoma or well-differentiated adenocarcinoma with MSI-H at the stage of chronic gastritis showed no evidence of *Helicobacter pylori* infection throughout the observation periods. These results indicate that MSI in biopsy specimens at the stage of chronic gastritis may predict the risk of the progression to adenoma and well-differentiated adenocarcinoma, and that *Helicobacter pylori* infection itself may not induce MSI directly in the gastric mucosa. © 2000 Cancer Research Campaign

**Keywords:** microsatellite instability; gastritis; gastric adenoma; gastric adenocarcinoma; *Helicobacter pylori*

Gastric cancer is still the second leading cause of cancer death in Japan, although its incidence and mortality tends to be decreasing. With recent development of minimum invasive surgery such as endoscopic mucosectomy and laparoscopic gastrectomy, it is crucial to find out lesions with gastric adenoma and adenocarcinoma at the early stage. However, no good predictable marker for development of adenoma and well-differentiated adenocarcinoma from non-malignant gastritis mucosa has been established.

Microsatellite instability (MSI) or replication error (RER) at microsatellite loci is mutator phenotype of genetic instability resulting from DNA mismatch repair failure of genes (Bronner et al., 1994; Leach et al., 1993; Parsons et al., 1993). MSI has been described in several cancers (Eshleman and Markowitz, 1995; Han et al., 1993) and is thought an important predisposition for human multistep carcinogenesis (Aaltonen et al., 1993; Ionov et al., 1993). Recent studies have demonstrated that MSI positive phenotypes were found in 20–40% of patients with gastric adenocarcinoma (Chong et al., 1994; Dos Santos et al., 1996) and also detected in gastritis mucosa adjacent to gastric adenocarcinoma (Semba et al., 1996). These results support that MSI may be an important and early event in gastric carcinogenesis of the intestinal type epithelium (Buonsanti et al., 1997; Chung et al., 1996). However, no direct evidence proving that well-differentiated adenocarcinoma and adenoma arise from intestinal metaplasia-associated gastritis mucosa with MSI has been reported.

The mechanism responsible for MSI in sporadic gastric cancer has not been elucidated. Gastric mucosal atrophy and intestinal metaplasia are considered as the earliest phenotypic changes in the cascade of events leading from normal mucosa to intestinal type gastric cancer (Correa, 1992; Rugge et al., 1994; Sipponen et al., 1984). *Helicobacter pylori* (*H. pylori*) infection has been reported as an important risk factor for gastric carcinogenesis (Nomura et al., 1991; Parsonnet et al., 1991). However, the association between *H. pylori* infection and gastric carcinogenesis is still hypothetical. In the present study, we first analysed mutations of microsatellite loci in biopsied paraffin-embedded tissues from patients retrospectively, to elucidate whether MSI leads to the progression from intestinal metaplasia-associated gastritis mucosa to gastric adenoma and adenocarcinoma. We also investigated whether *H. pylori* infection had a direct effect on MSI in the gastric mucosa of adenoma and adenocarcinoma.

*Received 5 January 1999*
*Revised 22 December 1999*
*Accepted 23 December 1999*

*Correspondence to:* T Hibi

### MATERIALS AND METHODS

### Samples

Biopsy specimens from 55 patients with chronic gastritis, 12 with gastric adenoma and 12 with well-differentiated, elevated type early gastric adenocarcinoma, located in the distal stomach, were obtained. Serial biopsied gastric tissues were obtained from 17 patients with gastric adenoma or well-differentiated adenocarcinoma to examine MSI phenotypes, endoscopic and histological findings retrospectively, for 1–7 years before the final diagnosis. To investigate the relationship between MSI phenotypes and mucosa of chronic gastritis adjacent to the carcinoma, specimens obtained by mucosectomy were examined in elevated-type early gastric adenocarcinoma. Histological examination confirmed that the adjacent mucosa was free of adenocarcinoma and adenoma. Tissues from non-tumour or non-inflammatory mucosa of the gastrointestinal tract, showing no dysplasia or metaplasia, were used as a control in analysis of microsatellite alterations. All samples were fixed in formalin and embedded in paraffin. All human studies are approved by the ethical review board of our institution.

### Analysis of microsatellite alterations

Microsatellite alterations on chromosomes 2p (D2S123), 3p (D3S1067), 5q (D5S409), 13q (D13S153), and 17p (TP53) were examined in paired tumour and normal DNA, as the alterations on those chromosomes have been demonstrated in gastric carcinomas. DNA was extracted by a standard proteinase K digestion and phenol/chloroform extraction procedure. The extracted DNA was amplified by PCR with an appropriate pair of biotinylated primers for each locus. The sequences of the primers used in this study were described previously (Tamura et al., 1995). Thirty-five cycles of PCR were performed using a denaturation step of 94°C for 0.5 min, an annealing step of 54°C for 1 min, and an elongation step of 72°C for 1 min in a total volume of 50 µl in 1 × PCR buffer containing 20 pM of each primer, 1 mM $MgCl_2$, 0.2 mM of each deoxynucleotide triphosphate, 0.5 units AmpliTaq DNA polymerase, and 100 ng genomic DNA. 45 µl of the PCR product were added with 5 µl of a 10 × gel loading buffer, and heated at 94°C for 3 min. Electrophoresis was performed on a 6% polyacrylamide gel containing 7 M urea at 30 W for 3.5–4 h. The gel was transferred to membrane, treated with streptavidin-biotin methods, and exposed to X-ray film for 30 min. Briefly, the biotinylated DNA, blotted onto a positive-charged nylon membrane was detected with a chemiluminescence method according to the manufacturer's recommended procedure (Imaging high, Chemilumi, Toyobo Co, Osaka, Japan). Biotin was linked to alkaline phosphatase with use of streptavidin. ALP activity was then detected with a chemiluminogenic substrate. The autoradiogram analysis of sample and control patterns was carried out.

MSI was determined by the mobility shift of PCR products. In gastric adenoma or adenocarcinoma with MSI, additional bands were found in the normal allele regions. On the basis of the number of microsatellite markers displaying instability per lesion, three groups were defined. MSI in two or more markers was classified as high-level MSI (MSI-H), MSI in one marker was interpreted as low-level MSI (MSI-L), and no MSI was defined as microsatellite stable (MSS) (Boland et al., 1998; Kim et al., 1999).

### Diagnosis of *H. pylori* infection

All gastric mucosal samples were stained with haematoxylin-eosin and microscopically evaluated by the pathologists for the presence of *H. pylori*. In patients with gastric adenoma or well-differentiated adenocarcinoma who showed MSI-positive phenotypes at the stage of chronic gastritis, the presence of *H. pylori* infection was also assessed by rapid urease test for gastritis mucosa and urea breath-test as described previously (Goodwin and Hendall, 1997).

### RESULTS

Mutations of five microsatellite loci were examined in paraffin-embedded sections from 55 patients with chronic gastritis. Only one of 55 patients (2%) with chronic gastritis, including 38 patients associated with intestinal metaplasia, revealed MSI-H phenotypes and the other 54 patients showed MSS phenotypes. Therefore, the frequency of MSI detected in chronic gastritis was extremely low.

We then analysed MSI-positive phenotypes using serial paraffin-embedded sections of biopsied gastric tissues in 17 patients with gastric adenoma or well-differentiated adenocarcinoma. We also examined endoscopic and histological findings in those patients retrospectively for 1–7 years before final diagnosis. MSI-positive phenotypes were demonstrated in six of 17 (35%) cases (Figure 1). One patient with gastric adenoma (case 7) and two patients with adenocarcinoma (cases 3 and 15) revealed MSI-H, and three other patients (cases 2, 10 and 11) showed MSI-L. Interestingly, all three patients who showed MSI-H and three who showed MSI-L at the time of final diagnosis revealed MSI-positive phenotypes in the specimens obtained at the stage of chronic gastritis. In two of three MSI-H cases, the identical MSI patterns at the stage of adenocarcinoma had been already observed at the stage of intestinal metaplasia-associated gastritis 1.5–7 years before the final diagnosis. In case 3, the sample of intestinal metaplasia-associated gastritis demonstrated MSI with an identical banding pattern as compared with that of *adenocarcinoma* (Figure 2A). In case 7, the sample of intestinal metaplasia-associated gastritis demonstrated MSI with a distinct banding pattern as compared with that of *adenoma* (Figure 2B).

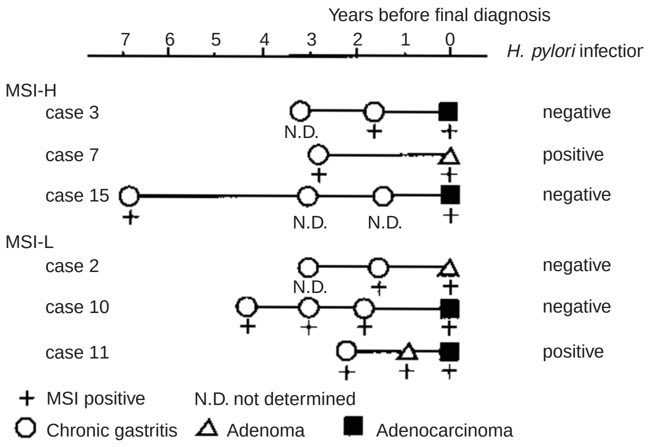

**Figure 1** Microsatellite instability, histological findings and *H. pylori* infection in serial biopsy specimens. Mutations of microsatellite loci in six patients with gastric adenoma and well-differentiated adenocarcinoma, who showed MSI-positive phenotypes in final diagnosis, were analysed retrospectively for 1.5–7 years before final diagnosis using serial gastric mucosa biopsied from the same area at different time points. Interestingly, all of six patients showing MSI including three high-level MSI (MSI-H) cases and three low-level (MSH-L) cases had already revealed MSI at the stage of chronic gastritis 1.5–7 years before the final diagnosis of gastric adenoma and adenocarcinoma. Two of three cases with gastric well-differentiated adenocarcinoma who showed MSI-H at the stage of chronic gastritis and in final diagnosis showed no evidence of *H. pylori* infection throughout the observation periods.

*© 2000 Cancer Research Campaign*

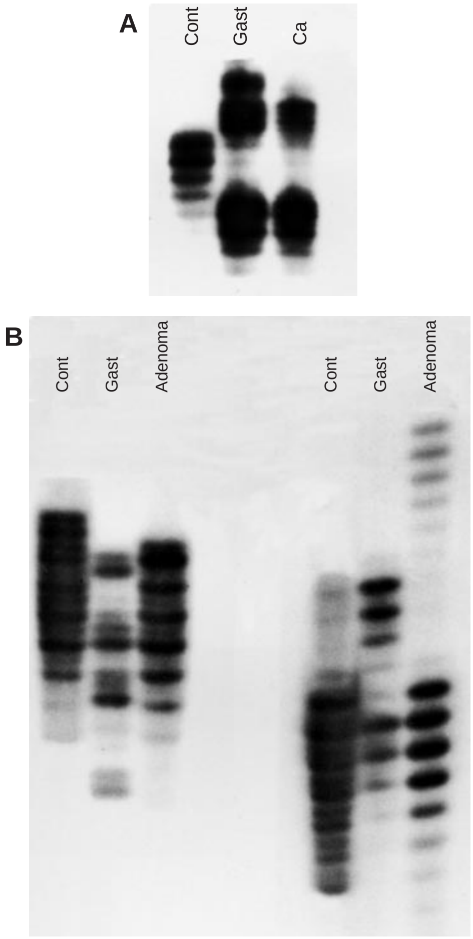

**Figure 2** Microsatellite instability banding patterns in cases 3 and 7 with gastric well-differentiated adenocarcinoma and adenoma, respectively, showed MSI-positive phenotypes in final diagnosis. (**A**) In case 3, identical MSI patterns at the stage of adenocarcinoma (Ca) had been already observed at the stage of intestinal metaplasia-associated gastritis (Gast) 1.5 years before the final diagnosis at two loci. Control DNA (Cont) was extracted from tissues of non-tumour or non-inflammatory mucosa of the gastrointestinal tract of the same patient. Primer used in this study was D13S153. (**B**) In case 7, MSI patterns at the stage of adenoma (Adenoma) were quite different from those in the sample of intestinal metaplasia-associated gastritis (Gast) at 2.5 years before final diagnosis at two different loci. Primers used in left and right panels were D5S409 and D13S153, respectively.

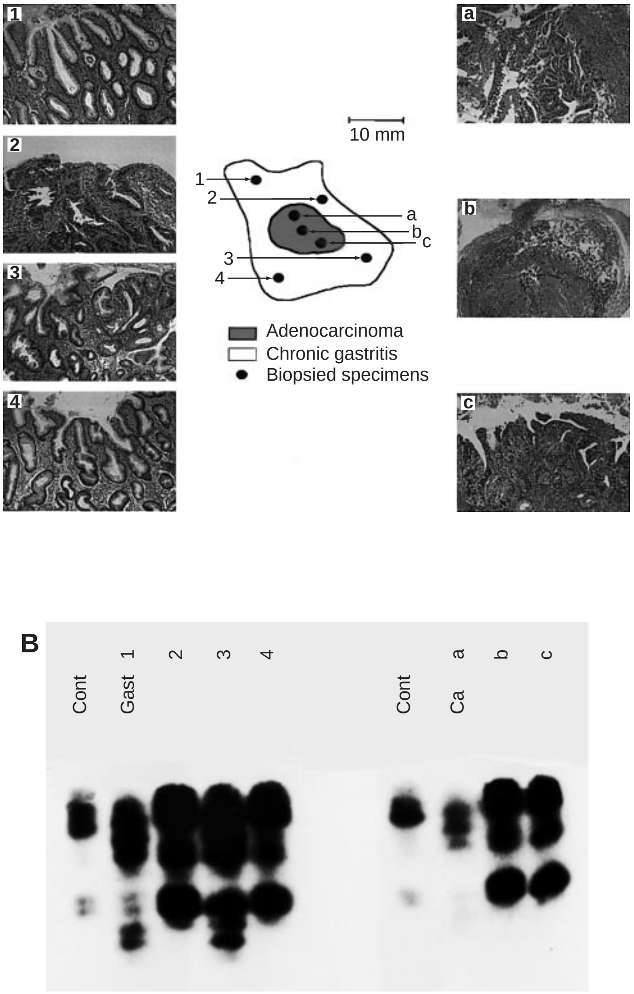

**Figure 3** (**A**) Schematic picture and histological findings of resected mucosa by endoscopic mucosectomy in case 15 with gastric well-differentiated adenocarcinoma who showed MSI-positive phenotypes in final diagnosis. Biopsied specimens were obtained from four different parts of the mucosa with gastritis (1, 2, 3 and 4) adjacent to elevated type early gastric adenocarcinoma (a, b and c). (**B**) MSI banding patterns, in four different parts of the mucosa with gastritis (Gast 1, 2, 3 and 4) adjacent to elevated type early gastric adenocarcinoma (Ca a, b and c), demonstrated MSI. Two different MSI banding patterns were observed in the mucosa of the gastritis and adenocarcinoma from the same patient. Primer used in this study was D3S1067.

We then examined MSI in the gastritis mucosa adjacent to the carcinoma to investigate the correlation between MSI-positive phenotypes and histological findings. In case 15, adjacent mucosa was resected by mucosectomy with elevated-type early gastric adenocarcinoma. All four specimens obtained from different parts of the mucosa with gastritis, within 10 mm away from gastric adenocarcinoma, demonstrated MSI (Figures 3A and 3B). Two different MSI banding patterns were observed in the adjacent gastritis mucosa and adenocarcinoma from the same patient.

To assess the effect of *H. pylori* infection on MSI, we then analysed MSI-positive phenotypes in 55 patients with chronic gastritis with or without infection. Only one case of 55 showed MSI, but this case showed no evidence of *H. pylori* infection. MSI was not found in any of 35 patients (10 with superficial gastritis, 11 with atrophic gastritis without intestinal metaplasia and 14 with atrophic gastritis with intestinal metaplasia) with *H. pylori* infection. Moreover, two of three cases with gastric adenoma or well-differentiated adenocarcinoma who showed MSI-H at the stage of chronic gastritis and in final diagnosis showed no evidence of *H. pylori* infection throughout the observation periods (Figure 1).

### DISCUSSION

In Japan, gastric lesions that Western pathologists classify into precancerous lesions are often diagnosed as gastric carcinoma (Schlemper et al., 1997). However, 2–4% of mucosal carcinomas and 14–20% of submucosal carcinomas that Western pathologists would diagnose as adenoma or dysplasia showed lymph-node metastasis (Sano et al., 1992). In addition, cumulative mortality

 *© 2000 Cancer Research Campaign*

rate of gastric cancer in Japan is extremely high as compared with that in Western countries. Therefore, diagnosis of gastric carcinoma at the early stage of the disease is crucial in Japan.

Several studies demonstrated that MSI-positive phenotypes were found in 20–40% of patients with gastric cancers, and more frequently in well-differentiated and antral gastric adenocarcinoma (Chong et al., 1994; Dos Santos et al., 1996). Moreover, MSI-positive phenotypes were also detected in intestinal metaplasia-associated gastritis mucosa adjacent to well-differentiated gastric adenocarcinoma (Semba et al., 1996). These results suggest that MSI may be an early event in gastric carcinogenesis, especially in the intestinal type cancer. However, there is no direct evidence supporting that well-differentiated adenocarcinoma and adenoma arise from chronic gastritis mucosa with MSI. In the present study, we demonstrated that the frequency of MSI in gastric adenoma and well-differentiated adenocarcinoma were almost the same as previously reported data. However, the frequency of MSI detected in chronic gastritis was extremely low.

We thus analysed MSI-positive phenotypes using serial sections of gastric mucosa biopsied from the same area at different time-points. To avoid a sampling error for doing biopsy in the gastritis mucosa at different time-points, we investigated correlation between MSI-positive phenotypes and histological findings of gastric adenocarcinoma and adjacent gastritis mucosa to the carcinoma. Several different areas in the adjacent gastritis mucosa within 10 mm of the cancer showed the same MSI as carcinoma. This result indicates that serial MSI-positive phenotype analysis of the same area at different time-points is feasible. MSI analysis using serial biopsied gastric tissues demonstrated that MSI-positive phenotypes were observed in six of 17 patients with gastric adenoma or well-differentiated adenocarcinoma and all three patients who showed MSI-H in final diagnosis revealed MSI at the stage of chronic gastritis. This result is in contrast to our finding that only 2% of chronic gastritis showed MSI. In two of three cases, the identical MSI patterns as at the stage of adenocarcinoma had been already observed at the stage of intestinal metaplasia-associated gastritis 1.5–7 years before the final diagnosis. These results favour the idea that mutations in microsatellite sequences in the biopsy specimens of gastric mucosa could predict precancerous lesions in patients with chronic gastritis. Chronic gastritis mucosa with intestinal metaplasia is considered as a precancerous lesion of intestinal type gastric cancer, and significantly associated with *H. pylori* infection (Nomura et al., 1991; Parsonnet et al., 1991). However, the association between the *H. pylori* infection and gastric carcinogenesis is still hypothetical. *H. pylori* infection induces active inflammation with neutrophilic inflammation, and also elicits chronic inflammation with infiltration of lymphocytes, macrophages/monocytes, and plasma cells in the lamina propria of the mucosa of gastric antrum (Correa, 1988). Oxygen free radicals produced by infiltrated cells could cause DNA damage to the adjacent cells (Baik et al., 1996). The increased cell turnover and impaired DNA repair in inflamed tissue is thought to increase the rate of establishment of DNA mutations. These results raise the possibility that *H. pylori* infection induces genetic instability in the gastric mucosa. To assess the effect of *H. pylori* infection on the MSI, we then analysed MSI in patients with chronic gastritis with or without infection. MSI-positive was not found in any of 35 patients with *H. pylori* infection. Moreover, two of three cases with gastric adenoma or well-differentiated adenocarcinoma who showed MSI-H at the stage of chronic gastritis and in final diagnosis showed no evidence of *H. pylori* infection throughout the observation periods. These results indicate that *H. pylori* infection is not correlated with MSI in the gastric mucosa and may not cause MSI directly in adenoma and well-differentiated adenocarcinoma.

Recent reports suggest that MSI-L does not qualify a gastric cancer as a mutator phenotype from a clinical point, because the occurrence of MSI at a few loci may represent the inherent instability of microsatellite markers or an abnormality of one of the DNA polymerases. MSI-H, described in hereditary nonpolyposis colorectal cancer-associated tumours (Boland et al., 1998), may indicate abnormalities of DNA mismatch repair genes in gastric cancer. However, there is considerable debate and controversy regarding the separation of tumours with low frequency versus those lacking MSI (MSS). In the present study, we observed that the frequency of MSI-L in non-malignant gastritis mucosa was extremely low and three cases with MSI-L in gastritis eventually developed into gastric adenoma and adenocarcinoma. Therefore, MSI-L phenotypes may be clinically significant in carcinogenesis of the stomach.

We are now doing prospective studies to prove that patients with intestinal metaplasia-associated gastritis showing MSI developed gastric adenoma and well-differentiated adenocarcinoma. We have found a patient with intestinal metaplasia-associated chronic gastritis who had MSI in the gastritis mucosa developed well-differentiated adenocarcinoma in the same area after a 3-year observation period (unpublished observation). Taken together with this finding, the present study indicates that microsatellite instability, both MSI-H and MSI-L, in the biopsy specimens of mucosa with chronic gastritis may identify patients at risk of developing gastric adenoma and well-differentiated adenocarcinoma.

### ACKNOWLEDGEMENTS

The authors would like to express thanks to Drs Yasushi Iwao, Haruhiko Ogata, Shigenari Hozawa and Nagamu Inoue for critical comments, Miss Motomi Yamazaki for technical assistance and Miss Reiko Fujisaki for manuscript preparation. This study was supported in part by grants-in-aid from the Japanese Ministry of Education, Culture and Science, the Japanese Ministry of Health and Welfare, Keio University and Keio Medical Foundation, Tokyo, Japan.

### REFERENCES

Aaltonen LA, Peltomaki P, Leach FS, et al (1993) Clues to the pathogenesis of familial colorectal cancer. *Science* **260**: 812–816

Baik SC, Youn HS, Chung MH, et al (1996) Increased oxidative DNA damage in *Helicobacter pylori*-infected human gastric mucosa. *Cancer Res* **56**: 1279–1282

Boland CR, Thibodeau SN, Hamilton SR, et al (1998) A national cancer institute workshop on microsatellite instability for cancer deletion and familial predisposition: development of international criteria for the determination of microsatellite instability in colorectal cancer. *Cancer Res* **58**: 5248–5257

Bronner CE, Baker SM, Morrison PT, et al (1994) Mutation in the DNA mismatch repair gene homologue hMLH1 is associated with hereditary non-polyposis colon cancer. *Nature* **368**: 258–261

Buonsanti G, Calistri D, Padovan L, et al (1997) Microsatellite instability in intestinal and diffuse-type gastric carcinoma. *J Pathol* **182**: 167–173

Correa P (1988) Chronic gastritis: a clinico-pathological classification. *Am J Gastroenterol* **83**: 504–509

Correa P (1992) Human gastric carcinogenesis, a multistep and multifactorial process. *Cancer Res* **52**: 6735–6740

© 2000 Cancer Research Campaign 

Chong J-M, Fukayama M, Hayashi Y, et al (1994) Microsatellite instability in the progression of gastric carcinoma. *Cancer* **54**: 4595–4597

Chung Y-J, Song J-M, Lee J-Y, et al (1996) Microsatellite instability-associated mutations associate preferentially with the intestinal type of primary gastric carcinomas in a high-risk population. *Cancer Res* **56**: 4662–4665

Dos Santos NR, Seruca R, Constancia M, et al (1996) Microsatellite instability at multiple loci in gastric carcinoma; clinicopathologic implications and prognosis. *Gastroenterology* **110**: 38–44

Eshleman JR and Markowitz SD (1995) Microsatellite instability in inherited and sporadic neoplasms. *Curr Opin Oncol* **7**: 83–89

Goodwin CS and Mendall MM (1997) *Helicobacter pylori* infection. *Lancet* **349**: 265–269

Han H-J, Yanagisawa A, Kato Y, et al (1993) Genetic instability in pancreatic cancer and poorly differentiated type of gastric cancer. *Cancer Res* **53**: 5087–5089

Ionov Y, Peinado MA, Malkhosyan S, et al (1993) Ubiquitous somatic mutations in simple repeated sequences reveal a new mechanism for colonic carcinogenesis. *Nature* **363**: 558–561

Kim JJ, Baek MJ, Kim L, et al (1999) Accumulated frameshift mutations at coding nucleotide repeats during the progression of gastric carcinoma with microsatellite instability. *Lab Invest* **79**: 1113–1120

Leach FS, Nicolaides NC, Papadopoulos N, et al (1993) Mutations of a mutS homolog in hereditary nonpolyposis colorectal cancer, *Cell* **75**: 1215–1225

Nomura A, Stemmermann GN, Chyou P, et al (1991) *Helicobacter pylori* infection and gastric carcinoma among Japanese Americans in Hawaii. *N Engl J Med* **325**: 1132–1136

Parsonnet J, Friedman GD, Vandersteen DP, et al (1991) *Helocobacter pylori* infection and the risk of gastric carcinoma. *N Engl J Med* **325**: 1127–1131

Parsons R, Li G-M, Longley MJ, et al (1993) Hypermutability and mismatch repair deficiency in RER+ tumor cells. *Cell* **75**: 1227–1236

Rugee M, Farinati F, Baffa R, et al (1994) Gastric epithelial dysplasia in the natural history of gastric cancer: a multicenter prospective follow-up study. *Gastroenterology* **107**: 1288–1296

Sano T, Kobori O and Muto T (1992) Lymph node metastasis from early gastric cancer: endoscopic resection of tumour. *Br J Surg* **79**: 241–244

Schlemper RJ, Itabashi M, Kato Y, et al (1997) Differences in diagnostic criteria for gastric carcinoma between Japanese and Western pathologists. *Lancet* **349**: 1725–1729

Semba S, Yokozaki H, Yamamoto S, et al (1996) Microsatellite instability in precancerous lesions and adenocarcinomas of the stomach. *Cancer* **77**: 1620–1627

Sipponen P, Kekki M and Siurala M (1984) Age-related trends of gastritis and intestinal metaplasia in gastric carcinoma patients and in controls representing the population at large. *Br J Cancer* **49**: 521–530

Tamura G, Sakata K, Maesawa C, et al (1995) Microsatellite alterations in adenoma and differentiated adenocarcinoma of the stomach. *Cancer Res* **55**: 1933–1936

 *© 2000 Cancer Research Campaign*
